# Seasonal Variation in Diatom Availability and Utilization by Juvenile Japanese Sea Cucumber *Apostichopus japonicus*

**DOI:** 10.3390/microorganisms14030677

**Published:** 2026-03-16

**Authors:** Takuma Matsumoto, Kohei Matsuno, Yuji Anaguchi, Nobuharu Inaba

**Affiliations:** 1 Civil Engineering Research Institute for Cold Region, Public Works Research Institute, Sapporo 062-8602, Japan; 2 Field Science Center for Northern Biosphere, Hokkaido University, Hakodate 041-8611, Japan; k.matsuno@fish.hokudai.ac.jp; 3 Faculty/Graduate School of Fisheries Science, Hokkaido University, Hakodate 041-8611, Japan; 4 Arctic Research Center, Hokkaido University, Sapporo 001-0021, Japan; 5 Ocean Construction Co., Ltd., Kurashiki 711-0924, Japan; yanaguchi@kaiyoh.com; 6 School of Biological, Earth and Environmental Science, Faculty of Science, University of New South Wales, Sydney, NSW 2052, Australia

**Keywords:** sea cucumber, *Apostichopus japonicus*, optimal food, diatom, *Tabularia*

## Abstract

Diatoms are considered an important food source for the commercially valuable sea cucumber *Apostichopus japonicus*. However, food sources for juvenile *A. japonicus* in the wild remain understudied, despite their importance for effective stock enhancement. In this study, seasonal diatom assemblages and viability in the feces of juvenile *A. japonicus* and in the feeding environments (biofilm, water column) were investigated using direct microscopy to evaluate diatom availability and utilization by the juveniles. Additionally, a laboratory feeding experiment was conducted to examine the digestibility of the predominant diatom (*Tabularia*) in the feces during the field survey. Field surveys have shown that diatoms are a major food item for juveniles, although their contribution varies seasonally with shifts in dominant food sources. Notably, *Tabularia* spp. occurred at a higher relative proportion in fecal assemblages than in the feeding environments and exhibited high mortality in the feces (96.3 ± 2.4%). Consistently, laboratory experiments showed marked degradation of intracellular contents in *Tabularia* sp. after excretion, supporting its digestibility. These findings have broadened our understanding of optimal food sources for *A. japonicus* juveniles as well as their feeding ecology in natural environments, informing the selection of more suitable diets and potentially enhancing stock enhancement practices.

## 1. Introduction

*Apostichopus japonicus* (Selenka, 1867), commonly known as the Japanese sea cucumber, is a temperate species primarily distributed across the northwestern Pacific, including China, Japan, the Korean Peninsula, and the Russian Far East [1]. It is one of the most economically and commercially valuable sea cucumber species globally, widely consumed as a delicacy, utilized in traditional medicine and as a source of bioactive compounds for health and functional foods [2,3]. The dried form, known as bêche-de-mer, can fetch prices as high as 3583 USD/kg in Chinese markets, surpassing those of many tropical sea cucumber species [4,5]. High market demand has driven intensive fishery efforts, leading to the overexploitation of sea cucumber species [6]. Over the past 30–50 years, *A. japonicus* populations have declined by at least 60%, and the species was listed as Endangered on the International Union for Conservation of Nature (IUCN) Red List in 2013 [7]. The natural recovery of depleted sea cucumber populations is expected to be slow, given their long lifespans and low recruitment rates [8]. Consequently, the release of hatchery-produced juveniles has been promoted as an effective strategy for restocking and restoring wild populations [9]. In Japan, where the catch of *A. japonicus* has declined by more than 30% over the past three decades [10], the large-scale production and release of hatchery-reared juveniles have been implemented nationwide [11]. However, in Hokkaido, the northernmost region of Japan, which accounted for 32.6% of the national sea cucumber catch in 2022 [12], landings have continued to decline since peaking in 2007 [13], despite more than 15 years of intensive juvenile release efforts [11]. High post-release mortality of *A. japonicus* juveniles is considered a major limiting factor in the success of these programs. It has been attributed to several causes, including insufficient food availability [14], dislodgement by wave action [15,16], and predation by invertebrates [17,18,19]. Therefore, improving our understanding of optimal habitat and feeding environments for juvenile *A. japonicus* is critical for enhancing the effectiveness of aquaculture and stock enhancement strategies.

*A. japonicus* is considered a deposit feeder that consumes organic matter, microorganisms (e.g., bacteria and diatoms), and fragments of macrophytes [20]. Its food sources have been extensively investigated using a variety of approaches, including traditional direct microscopy [21,22], fatty acid biomarkers [23], stable isotope analysis [24,25], and molecular biological techniques [26,27,28]. These previous studies on the food sources have identified diatoms as one of the major dietary components in natural environments. However, these studies have predominantly focused on adult individuals, largely because juveniles occur at low densities and exhibit cryptic behavior in the wild [29]. As a result, many aspects of the ecology of juvenile *A. japonicus*, including their feeding habits, remain poorly understood. Notably, juveniles are often found in habitats distinct from those occupied by adults [30], suggesting that their optimal food sources may differ. Despite limited knowledge of juvenile diets in the wild, hatchery-reared juveniles are commonly fed commercial diets and benthic diatoms that spontaneously grow on substrates within nursery tanks [11,31]. Moreover, several studies have demonstrated that specific diatom species can enhance the growth of juvenile *A. japonicus* under laboratory conditions [32,33,34]. Collectively, these findings suggest that diatoms may also serve as an important food source for juvenile *A. japonicus* in natural environments. In contrast, diatoms are generally considered difficult for sea cucumbers to digest because of their rigid siliceous cell walls, known as frustules, which act as an effective barrier against predation [35,36]. Although sea cucumbers lack specialized organs capable of physically breaking down or chemically digesting these structures [37], the viability of diatom cells after ingestion by *A. japonicus* has received little attention [38], despite the frequent occurrence of diatoms in the gut contents and feces of sea cucumbers. If diatom cells remain structurally intact and viable after ingestion, this would indicate limited digestion of diatoms by *A. japonicus*, suggesting that diatoms may not represent an optimal or efficient food for this species. To the best of our knowledge, no previous studies have directly examined the viability of diatom cells ingested by juvenile *A. japonicus* under natural or semi-natural conditions.

In this study, we seasonally investigated diatom communities in both the feeding environments and the feces of hatchery-produced juvenile *A. japonicus* released into artificial reefs installed in a fishing port along the coast of Hokkaido, Japan, using direct microscopy. We compared the taxonomic composition and viability of diatoms in the feeding environments (the sympatric biofilms and the surrounding water column) with those in the feces of juvenile *A. japonicus* to determine whether the proportion of viable diatom cells was reduced after ingestion and to identify diatom species that were preferentially utilized by the juveniles. In addition, to complement the field survey, a preliminary laboratory feeding experiment was conducted to examine the digestibility of diatom species that were predominantly detected as non-viable cells in the juvenile feces during the field survey, thereby providing insight into the mechanisms of diatom digestion by juvenile *A. japonicus*.

## 2. Materials and Methods

### 2.1. Field Survey

The field survey was carried out within a fishing port (depth of 4–5 m) located on the coast of Oshima Peninsula, southwestern Hokkaido, Japan. In the fishing port, release of *A. japonicus* juveniles has been practiced by local fishers. Artificial intermediate sea cucumber reefs were deployed on the fishing port seafloor (41°27′44″ N, 140°14′52″ E), containing scallop shells as attachment substrates for released *A. japonicus* juveniles. Hatchery-produced juveniles of *A. japonicus* were released into the artificial reefs in December 2018. The juveniles used in the field survey were purchased from Hokkaido Aquaculture Promotion Corporation.

**Figure 1:**
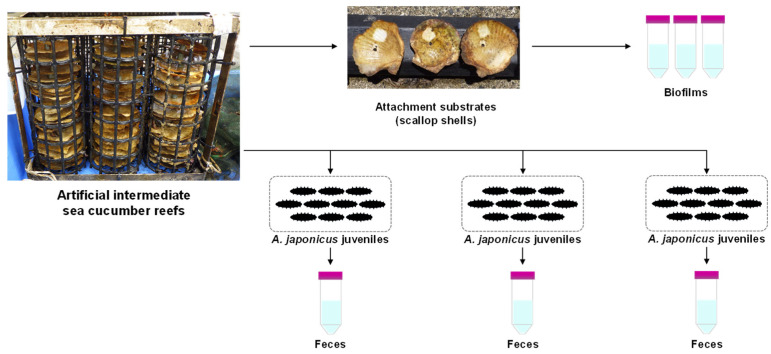
Sampling of biofilms and feces of *A. japonicus* juveniles from artificial intermediate sea cucumber reef.

The artificial reefs were landed every three months from March to December 2019. After landing the artificial reefs, scallop shells (*n* = 3) were randomly collected from the reefs, and biofilms formed on the surface of each shell were scraped off with a sterile toothbrush from a certain area of the substrates (5.29 cm^2^) and individually suspended in a specific volume of artificial seawater (ASW) to obtain biofilm samples (Figure 1). Sampling of biofilms was conducted immediately after the collection of the scallop shells. Additionally, juveniles of *A. japonicus* (<10 g) were randomly collected from three reefs (maximum 10 individuals per reef). The juveniles collected from each reef were gently cleaned with ASW and kept individually in a bottle filled with ASW until defecation. Feces from all the juveniles of each reef were collected with a clean pipette and suspended in sterile ASW to obtain a fecal sample for each reef (Figure 1). Furthermore, the surface (0.5 m below the surface) and bottom (0.5 m above the seafloor) seawaters in the proximity of the artificial reefs were collected with a Van Dorn water sampler. The vertical profiles of hydrographic parameters in the sympatric water column (water temperature, salinity, and turbidity) were also determined with a RINKO-profiler (JFE Advantech, Nishinomiya, Japan).

Subsamples of seawater, biofilms, and feces were filtered using Whatman GF/F filters. The filters were immersed in 90% acetone to extract pigments under cool and dark conditions, and chlorophyll *a* (Chl. *a*) and pheophytin (Pheo) concentrations were determined fluorometrically (Trilogy, Turner Designs, Inc., San Jose, CA, USA) by measuring fluorescence according to Holm-Hansen et al. [39]. Another subsample of seawater, biofilms, and feces for diatom analysis was fixed with 25% glutaraldehyde (1% final concentration) and stored in a cold, dark environment until the analysis. For diatom analysis, an aliquot of each sample was mounted on a glass slide, and diatoms (>10 µm) were enumerated and identified to the genus level under an inverted optical microscope (600×, Eclipse, Nikon, Tokyo, Japan). Because cell densities varied among samples, subsamples of appropriate volume were taken depending on the cell density to obtain a countable number of cells (typically >300 cells) for microscopic enumeration. During enumeration, viable cells (retaining chloroplasts and intracellular contents) and non-viable cells (empty or partially broken) were counted separately to determine diatom cell viability in each sample. In the present study, a partially broken cell was defined as a cell with a partially damaged frustule but retaining sufficient morphological features to allow identification to the genus level. Cell counts were performed once per sample. Diatom genera were identified primarily based on Jin et al. [40] and Hasle and Syvertsen [41], with additional taxonomic references consulted for certain genera, particularly *Tabularia* [42,43,44].

### 2.2. Laboratory Feeding Experiment

**Figure 2:**
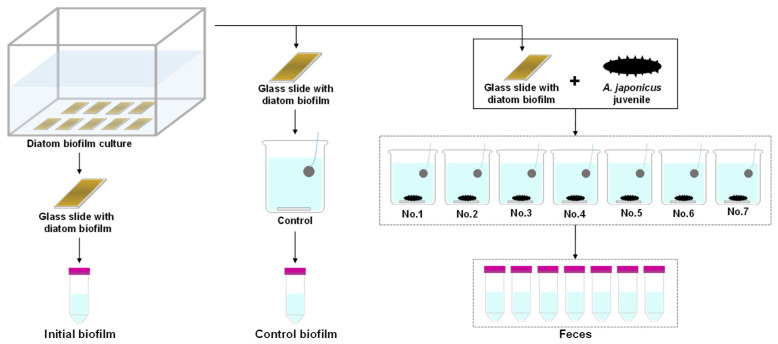
Workflow of sampling biofilms (initial and control) and feces of *A. japonicus* juveniles in the laboratory feeding experiment.

Given that the periphytic diatom genus *Tabularia* was detected as one of the dominant diatom taxa in the fecal samples of the field survey, *Tabularia* sp. was isolated from the biofilm collected at the field study site and established as a monoculture for a subsequent experiment. The subculture was maintained at 15 °C in Daigo’s IMK medium (Shiotani MS, Amagasaki, Japan), which supports stable growth of a wide range of marine microalgae, including diatoms [45,46], supplemented with silicate under a 14 h light/10 h dark photoperiod until the feeding experiment. The medium was prepared using artificial seawater (Marine Art SF-1, Tomita Pharmaceutical, Naruto, Japan). For diatom feed preparation, 10 mL of the subculture was dispensed into a plastic container filled with 400 mL of the same medium, with clean glass slides placed at the bottom (Figure 2). The container was then incubated for 6–10 days under the same conditions mentioned above until a dense *Tabularia* sp. biofilm covered the slide surfaces. *A. japonicus* juveniles for the experiment were purchased from Sanpou Co., Ltd. (Hokkaido, Japan), acclimated to laboratory rearing conditions (13 ± 1 °C under a 12 h light/12 h dark photoperiod with continuous aeration) for 7 days, and starved for 2 days prior to the experiment. To determine the initial diatom density on glass slides, one slide was randomly selected prior to the feeding experiment, and the biofilm was entirely scraped off using a clean cell scraper and suspended in ASW for analysis. Seven juveniles (average wet weight: 0.62 ± 0.30 g) were randomly selected and placed individually in 500 mL glass beakers filled with GF/F-filtered seawater, each containing one glass slide with *Tabularia* sp. biofilm. Another beaker without juvenile *A. japonicus* was also prepared as a control to assess the effects of experimental conditions on diatom viability. The feeding experiment was conducted for 24 h at the rearing conditions described above. After 24 h, the slide and *A. japonicus* were removed from each beaker, and all fecal pellets were carefully collected with a clean pipette. Fecal pellets collected from each beaker were then individually suspended in ASW to obtain fecal samples. The entire biofilm on the glass slide in a control beaker was also collected with a clean cell scraper and suspended in ASW. The experiment was repeated three times. Chl. *a* content in the biofilm and feces was measured using the same protocol as that used for the field pigment analysis. Subsamples for diatom composition and viability were fixed with 25% glutaraldehyde (1% final concentration) and stored in a cool, dark environment until analysis. Diatom cell densities in the biofilm and feces of the juveniles were determined under an inverted optical microscope (600×, IX73, Olympus, Tokyo, Japan), and cell viability was assessed based on the presence of chloroplasts and intracellular contents.

### 2.3. Statistical Analysis

Statistical analyses were performed using R (version 4.5.0). Differences in the proportion of non-viable cells among the water column, biofilms, and feces of the field survey, and differences in Chl. *a* content and the proportion of *Tabularia* sp. among the biofilms and the feces of the laboratory experiment were assessed by one-way ANOVA followed by Tukey–Kramer post hoc tests. Assumptions of normality and homogeneity of variances were examined using the Shapiro–Wilk and Levene’s tests, respectively, and data were log-transformed when necessary. A significance level of *p* < 0.05 was considered statistically significant.

## 3. Results

### 3.1. Hydrographic Conditions

**Figure 3:**
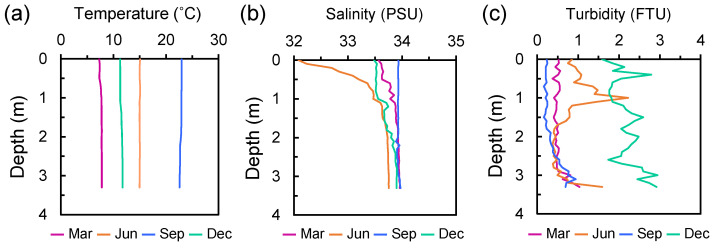
The vertical profiles of (**a**) water temperature, (**b**) salinity, and (**c**) turbidity in the water column around the artificial reefs from March to December 2019.

Water temperature was nearly uniform throughout the water column across all seasons (Figure 3a). The mean temperatures were 7.7 ± 0.16 °C in March, 15.0 ± 0.03 °C in June, 22.8 ± 0.15 °C in September, and 11.5 ± 0.19 °C in December 2019. Salinity of the water column was generally within the range of 33.5 to 34 over the study period, except in June, when relatively low-salinity water (32.1–33.4) was observed at depths shallower than 1 m (Figure 3b). Turbidity was relatively high throughout the water column in December, with a mean value of 2.19 ± 0.36 FTU, although it generally ranged below 1, with mean values of 0.52 ± 0.13 µg L^−1^ (March), 0.81 ± 0.44 µg L^−1^ (June) and 0.36 ± 0.21 µg L^−1^ (September), except in June, when relatively high turbidity (>1) was observed around a depth of 1.0 m (Figure 3c).

### 3.2. Seasonal Diatom Assemblages and Viability

**Figure 4:**
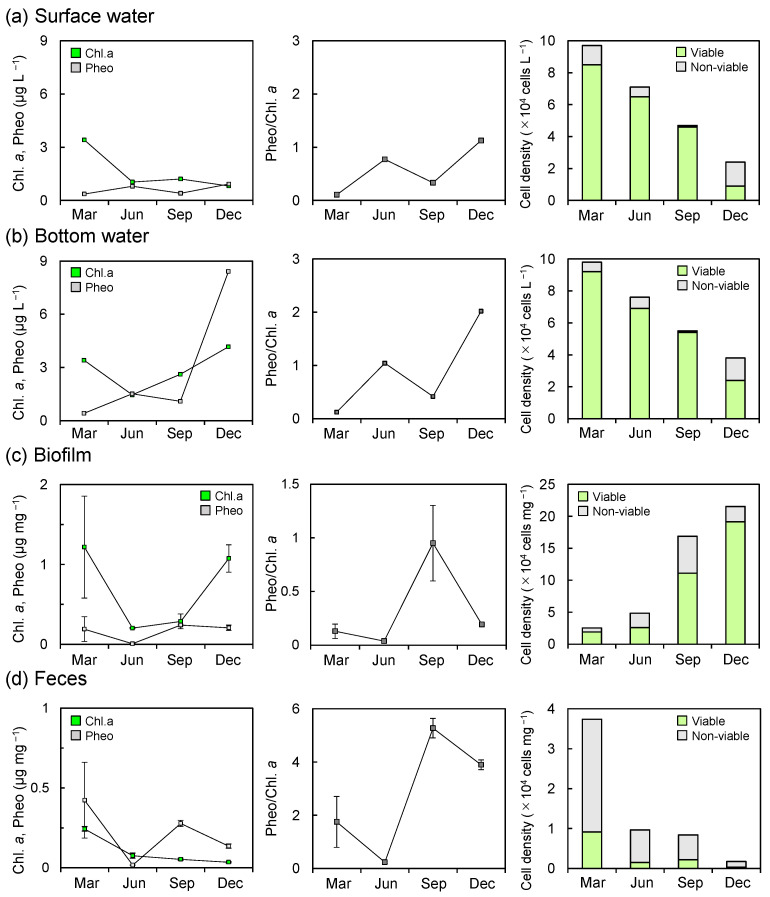
Seasonal changes in pigment concentrations (Chl. *a* and Pheo), Pheo/Chl. *a* ratios, and diatom densities in (**a**) surface and (**b**) bottom waters, (**c**) biofilms, and (**d**) feces of juvenile *A. japonicus* from March to December 2019.

**Figure 5:**
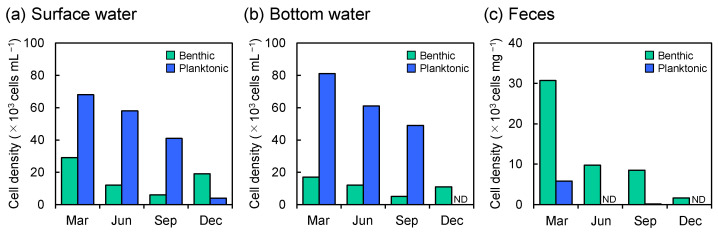
Seasonal changes in benthic and planktonic diatom cell densities of (**a**) surface and (**b**) bottom waters, and (**c**) feces of juvenile *A. japonicus* from March to December 2019. ND indicates not detected in the sample.

In the surface water, Chl. *a* concentrations showed an overall decreasing trend during the study period, ranging from 0.8 to 3.4 µg L^−1^, with a pronounced peak in March (Figure 4a). In contrast, Pheo concentrations steadily increased over time, ranging from 0.37 to 0.92 µg L^−1^, with minor peaks observed in June and December. In the bottom water, Chl. *a* concentrations declined from March to June and subsequently increased toward December, ranging from 1.46 to 4.16 µg L^−1^, with the lowest and highest values recorded in June and December, respectively (Figure 4b). Although Pheo concentrations in the bottom water exhibited a seasonal pattern similar to that in the surface water, they reached a markedly higher value of 8.41 µg L^−1^ in December. From June to December, Chl. *a* and Pheo concentrations in the bottom water were 1.4–5.1 and 1.9–9.1 times higher, respectively, than those in the surface water, whereas the concentrations were comparable between the two layers in March. The Pheo/Chl. *a* ratios in both surface and bottom waters exhibited similar seasonal trends, increasing from 0.11 to 1.13 and from 0.12 to 2.02, respectively, with distinct peaks in June and December (Figure 4a,b). Diatom cell densities in both surface and bottom waters progressively decreased from March to December, ranging from 2.4 × 10^4^ to 9.7 × 10^5^ cells L^−1^ and 3.8 × 10^4^ to 9.8 × 10^5^ cells L^−1^, respectively (Figure 4a,b). The proportion of non-viable diatom cells was relatively low from March to September, ranging from 2.1% to 12.4% in the surface water and from 1.8% to 9.2% in the bottom water, but increased sharply in December, reaching 62.5% and 36.8%, respectively. The dominant diatom species in the water column exhibited clear seasonal variations. From March to September, planktonic diatoms prevailed throughout the water column, with *Thalassiosira* spp. dominant in March (59.8–67.3%), *Leptocylindrus* spp. in June (32.9–54.3%), and *Chaetoceros* spp. in September (48.1–70.2%). In December, in contrast, benthic diatoms, including *Navicula* spp. and *Tabularia* spp., were more abundant in the water column (Table 1). Planktonic diatom cell densities were 2.3–6.8 and 4.8–9.8 times higher than benthic diatoms from March to September, but decreased markedly in December (Figure 5a,b).

Chl. *a* concentrations in the biofilm exhibited higher values of 1.22 ± 0.64 µg mg^−1^ and 1.08 ± 0.17 µg mg^−1^ in March and December, and lower values of 0.20 ± 0.01 µg mg^−1^ and 0.29 ± 0.09 µg mg^−1^ in June and September. The Pheo concentrations ranged from 0.19 ± 0.16 to 0.24 ± 0.01 µg mg^−1^ over the study period, except in June, when the remarkably low value of 0.008 ± 0.003 µg mg^−1^ was recorded (Figure 4c). The biofilm Pheo/Chl. *a* ratio ranged below 1 (0.04 ± 0.01–0.95 ± 0.35), with the lowest value in June and the highest in September, while relatively low values of 0.13 ± 0.07 and 0.19 ± 0.01 were observed in March and December, respectively (Figure 4c). In the biofilm, diatom cell density increased progressively over the study period, ranging from 3.9 × 10^4^ to 3.1 × 10^5^ cells mg^−1^, in contrast to the water column, which showed the opposite seasonal trend (Figure 4c). The proportion of non-viable cells in the biofilm was consistently lower than that of viable cells, ranging from 11.3% to 46.0%, with an average of 29.1 ± 12.7%. The biofilm diatom community consisted exclusively of benthic genera, with *Amphora* spp., *Cocconeis* spp., *Navicula* spp., *Nitzschia* spp., *Opephora* spp., and *Tabularia* spp. consistently observed, representing mean proportions of 8.4 ± 2.4%, 11.8 ± 5.8%, 12.1 ± 2.6%, 25.3 ± 11.2%, 28.4 ± 11.2%, and 12.0 ± 6.6%, respectively (Table 1).

In the feces, Chl. *a* concentrations consistently declined from 0.24 ± 0.01 to 0.04 ± 0.003 µg mg^−1^ throughout the study period (Figure 4d), whereas Pheo concentrations seasonally fluctuated, ranging from 0.02 ± 0.001 to 0.42 ± 0.24 µg mg^−1^, with the highest value in March and the lowest in June. The Pheo/Chl. *a* ratio in the feces ranged above 1 (1.7 ± 1.0 –5.3 ± 0.4) during the study period, with a peak in September, except for June, when the lowest value of 0.2 ± 0.04 was observed (Figure 4d). In the feces of *A. japonicus* juveniles, diatom cell density continuously declined over the study period from 3.7 × 10^4^ to 1.7 × 10^3^ cells mg^−1^, with a distinct peak in March (Figure 4d). In contrast to the feeding environments, non-viable diatom cells consistently outnumbered viable cells in the feces, with an average proportion of 84.1 ± 5.8% over the study period. The mean proportion of non-viable cells in the feces was higher than in both surface and bottom waters, although a significant difference was detected only between the feces and the water column (*p* < 0.05). Benthic diatoms, *Navicula* spp. and *Tabularia* spp., were also dominant in the feces, with higher average proportions of 19.8 ± 3.2% and 27.1 ± 11.1%, respectively (Table 1). *Tabularia* spp. exhibited higher mortality (96.3 ± 2.4%) throughout the study period compared with *Navicula* spp. (82.3 ± 6.9%). Although benthic diatoms dominated in terms of cell density, planktonic diatoms such as *Thalassiosira* spp. and *Chaetoceros* spp., which were dominant in the water column during March and September, also appeared in the feces during the same periods with compositions of 14.4% and 1.8%, respectively, while they were not detected in the feces in June and December (Figure 5c, Table 1).

### 3.3. Laboratory Feeding Experiment

**Figure 6:**
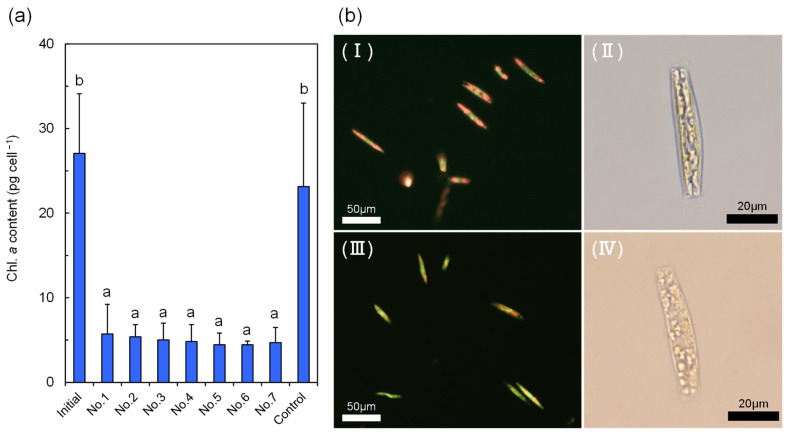
(**a**) Mean Chl. *a* content per *Tabularia* sp. cell in biofilms (initial and control) and in feces from *A. japonicus* juveniles (No. 1–7) in the laboratory feeding experiment. Different letters indicate a significant difference (*p* < 0.05). (**b**) Photomicrographs of *Tabularia* sp. cells in (**I**,**II**) initial biofilm and (**III**,**IV**) feces of *A. japonicus* juveniles.

All experimental juveniles exhibited active feeding behavior, spreading and retracting their tentacles over a glass slide to ingest the *Tabularia* sp. biofilm. Chl. *a* content and the proportion of viable *Tabularia* sp. cells did not differ significantly between the initial and control biofilms, with averages of 27.1 ± 7.0 pg cell^−1^ and 23.1 ± 9.9 pg cell^−1^, and 97.3 ± 1.4% and 95.9 ± 4.4%, respectively (Figure 6a). In contrast, Chl. *a* content per *Tabularia* sp. cell in juvenile feces was significantly lower than in the initial and control biofilms (*p* < 0.05), ranging from 4.4 ± 0.4 to 5.7 ± 3.5 pg cell^−1^ (Figure 6a), and chlorophyll autofluorescence was also observed to be attenuated in fecal *Tabularia* sp. cells (Figure 6b). Although the proportion of *Tabularia* sp. cells with remaining intracellular contents in the feces was comparable to that in the biofilms, averaging 89.6 ± 6.9–98.3 ± 0.4%, partial disintegration of intracellular contents was also evident in fecal cells (Figure 6b).

## 4. Discussion

Diatoms are considered a major food component for the Japanese sea cucumber *A. japonicus* [21,22,23,24,25,26,27,28], although the food sources of its juveniles in the wild remain poorly understood. In this study, diatoms were consistently present in the feces of juvenile *A. japonicus* during the field survey. However, fecal diatom density showed an opposite trend to that of the co-occurring biofilm, which has been considered an optimal food source for *A. japonicus* [47]. Specifically, fecal diatom density declined steadily over the study period, whereas biofilm diatom density increased progressively. In March, fecal diatom density was highest, despite the fact that the biofilm exhibited the lowest diatom density. On the other hand, the highest Chl. *a* concentrations were recorded in the surface water during this period. Furthermore, diatom density in the water column (both the surface and bottom waters) also peaked and was dominated by *Thalassiosira,* a major spring-bloom-forming diatom along the coasts of Hokkaido [48,49,50]. Consistent with this, the abundance of planktonic diatoms in the feces was also highest in March, with *Thalassiosira* spp. accounting for 14.4% of the assemblage. In fact, Thalassiosiraceae diatoms were most abundant in the gut contents of adult *A. japonicus* in spring on the coast of China as well [27]. Additionally, Yamazaki et al. [28] also indicated the food preference of the sea cucumber for planktonic diatoms (Chaetocerotaceae) in April. Notably, the density of benthic diatoms in the water column was also highest during the same period. These results suggest that increased diatom availability from the ambient water column during this period may have resulted in luxury feeding behaviour [51], leading to the highest cell density in the feces. In contrast, from June to September, planktonic diatoms were not detected or were scarce in the feces of juvenile *A. japonicus*, despite their relatively high abundance in the ambient water columns. In June, biofilm diatom density was relatively low; however, the Pheo/Chl. *a* ratio, an indicator of the physiological condition of autotrophs [52], was markedly low (0.04 ± 0.01), suggesting the biofilms were dominated by fresh, actively growing photosynthetic biomass likely from non-diatom autotrophs. Consistently, the fecal Pheo/Chl. *a* ratio was also minimal (0.24 ± 0.04). Moreover, diatom density in the feces was low, and the fecal assemblage was dominated by non-viable cells. Together, these findings imply that juveniles may have fed on photosynthetic biomass mainly derived from non-diatom autotrophs. Supporting the above interpretation, Liu et al. [47] reported that filamentous green algae proliferated in co-occurring biofilm on artificial reefs in a culture pond for *A. japonicus* during June–August, and stable isotope analysis indicated that chlorophytes constituted the primary food source during this period [23]. Correspondingly, in the present study, the surfaces of the attachment substrates were covered with green algae in June. In addition, the proliferation of brown macroalgae (*Undaria pinnatifida* and *Saccharina* kelps) was observed at the study site during the same period. Thus, fresh algal biomass other than diatoms likely contributed substantially to the juvenile diets in June. However, as suggested by David et al. [53], fresh macroalgae themselves may not be fully utilized by juveniles, which may have led to the lowest fecal Pheo/Chl. *a* ratio observed in June. In September, in contrast, the highest Pheo/Chl. *a* ratio in the biofilm (0.95 ± 0.35) was observed, despite the second-highest abundance of diatoms, suggesting dominance of physiologically degraded or senescent algal biomass. Consistently, the feces also exhibited the highest Pheo/Chl. *a* ratio (5.3 ± 0.37) during this period, indicating that *A. japonicus* juveniles ingested degraded organic material along with diatoms. During the same period, detritus derived from the sympatric dominant brown macroalgae was likely provided abundantly to the surrounding environments, as decomposition of these macroalgae generally progresses from August to October along the coasts of northern Japan, including Hokkaido [54,55,56]. Previous studies have shown that macroalgal detritus can serve as an important food source for *A. japonicus* [23,27]. In agreement with this, Laminariaceae (including the genus *Saccharina*) has been reported to be abundant in the feces of *A. japonicus* in September in a coastal culture pond in Hokkaido [28]. Collectively, although potential food components other than diatoms were not investigated in this study, these findings suggest that other photosynthetic organisms and their detritus served as a main food source for the juveniles in June and September. Notably, in December, the feces exhibited the lowest diatom density, whereas the highest diatom density was observed in the biofilm. The water column in December also exhibited the lowest diatom density. Moreover, the highest Pheo/Chl. *a* ratio in the water column (1.13–2.02), along with elevated turbidity and the highest Pheo concentrations, indicated that detrital particles containing degraded pigments were dominant rather than fresh phytoplankton biomass in the water column. The study site is influenced by two small rivers that transport terrestrial-derived materials, such as woody debris and leaf litter, into the fishing port. During the sampling in December, precipitation over the study area temporarily increased (25 mm d^−1^) [57], which likely led to an increase in discharge from the adjacent rivers. Consistently, feces in December were more unformed than in other seasons, and terrestrial leaf-like fragments were often observed. Similar patterns have been reported from an island off the coast of China, where terrestrial plant materials were also predominant in the gut contents of *A. japonicus* in December [27]. These observations suggest that the food sources of juvenile *A. japonicus* in December were derived from the ambient water column, containing abundant allochthonous terrestrial organic matter. Many sea cucumber species, including *A. japonicus* adults, are known to selectively ingest organically rich particles from their feeding environments [58,59]. While juvenile-stage evidence remains scarce, juveniles of *Australostichopus mollis*, a closely related species in the same family as *A. japonicus*, can also select organic particles from ambient sediments [60]. In contrast, direct evidence for this behavior in juvenile *A. japonicus* is lacking. In this study, the field survey indicated that diatoms are a fundamental component of the diet of wild juvenile *A. japonicus*. However, their relative contribution varied seasonally, reflecting a shift in where juveniles primarily fed—between suspended particles in the water column and organic-rich biofilm—likely mediated by selective ingestion of organic matter.

In the field survey, the benthic diatoms *Tabularia* spp., the most dominant taxa in the fecal diatom assemblages, were constantly more abundant in the feces than in the co-occurring biofilm, regardless of the seasonal variations in diatom ingestion by juvenile *A. japonicus*. Passy [61] classified diatom species in stream biofilms into three ecological guilds: low profile, high profile, and motile guild, and defined the high profile guild as “species of tall stature, including erect, filamentous, branched, chain-forming, tube forming, stalked, and colonial centrics”. *Tabularia* diatoms also exhibit an erect growth form, categorized as the high profile guild [62]. In general, diatom species that belong to the high profile guild are considered vulnerable to disturbance, including grazing, due to their ability to locate beyond the surface of a biofilm [61,63]. Thus, *Tabularia* diatoms may be easily scraped off by *A. japonicus* juveniles, resulting in the higher proportion in the feces. Furthermore, *Tabularia* spp. were not only dominantly and consistently contained in the feces during the field survey, but also exhibited high mortality, with a mean of 96.3 ± 2.4%. In the laboratory feeding experiment, fecal *Tabularia* sp. cells also showed statistically significant decreases in cellular Chl. *a* content and partial decomposition of intracellular contents compared with those in the diatom biofilm diet. These results therefore suggest that *Tabularia* diatoms are a valuable food option for *A. japonicus* juveniles because of their availability and digestibility.

During the field survey, fecal diatom assemblages contained a markedly higher proportion of non-viable cells (78.3–92.4%) than those in the surrounding feeding environments (2.1–62.5%, 1.8–36.8%, and 11.3–46.0% in surface water, bottom water, and biofilm, respectively), consistent with observations in other holothurian sea cucumbers in which the ratio of dead diatom cells is higher compared with that of adjacent sediments after ingestion [64,65]. Diatom frustules are thought to serve a protective function against predators [35,36]. Some zooplankton, including copepods and euphausiids, have evolved specialized structures that can break these hard siliceous frustules, allowing them to efficiently assimilate diatom cell contents [66,67]. *A. japonicus* is thought to rely primarily on mechanical trituration [32] because sea cucumbers lack specialized grinding structures or digestive glands specialized for breaking or decomposing such hard shells [37]. However, no obvious broken frustules were found in the feces of juvenile *A. japonicus* in the laboratory experiments, and only a few were observed in the field survey, despite reports of extensive frustule breakage in some species (e.g., *Holothuria theeli*) [65]. These findings suggest that intracellular contents may be processed largely without noticeable shell fragmentation. In contrast to the field survey, a higher proportion of *Tabularia* sp. cells in laboratory feces retained intracellular contents to some extent. This contrast in the proportion of completely empty frustules might be attributed to the following differences between the laboratory and field conditions. The mean percentage of non-viable *Tabularia* spp. cells in the sympatric (field) biofilm was 31.1 ± 24.6%, which was considerably higher than that in the laboratory biofilm (2.7 ± 1.4%). Consequently, the biofilm in the natural environment likely contained a greater abundance of degraded diatom cells than the biofilm under laboratory conditions, which may have contributed to the higher mortality observed in the field experiment. The presence of mineral particles in the diet is known to enhance food digestibility by increasing the residence time of ingested material in the gut and facilitating nutrient absorption [33]. Indeed, the growth performance of *A. japonicus* juveniles improves when a certain proportion of sea mud is incorporated into a diatom-based diet, compared with diets composed solely of diatoms [32]. Additionally, the experimental juveniles were fed a formulated feed for sea cucumbers (Namako Growth, Nosan Corporation, Yokohama, Japan) until the feeding experiment. Recent studies indicate that the gut microbiota of *A. japonicus* changes significantly in response to dietary shifts [68,69], while the gut microbiome is reported to play a significant role in food digestion in sea cucumbers, including *A. japonicus* [70,71,72]. Some of the dominant gut bacteria of *A. japonicus* have the capacity to degrade algal polysaccharides [71,72]. In addition, although endogenous digestive enzymes of *A. japonicus* may contribute to the digestion of organic materials associated with diatoms, the gut microbiome assists in the degradation of polysaccharides that *A. japonicus* cannot efficiently digest alone [71]. Although the gut microbiome of *A. japonicus* juveniles was not investigated in this study, these findings collectively suggest that gut bacteria may facilitate the digestion of intracellular contents of diatoms in juveniles.

Differences in frustule robustness among diatom taxa could affect post-ingestion outcomes. During the field survey, *Thalassiosira* spp., which have relatively robust frustules [35], were frequently observed in the feces in March, accounting for 14.4% of the fecal assemblage, and often retained intracellular contents and intact frustules (65.1%). Conversely, planktonic diatoms with generally weaker silicification, such as *Chaetoceros* and *Leptocylindrus* [41,73], were scarce or undetectable in feces, despite dominating the sympatric water column (e.g., *Chaetoceros* spp. accounted for 48.1–70.2% of the water column in September, but only 1.8% of fecal samples). Observed *Chaetoceros* spp. cells in feces were entirely non-viable, with partially broken frustules and lacking their intracellular contents. Notably, this low representation of the genus *Chaetoceros* in microscopy-based fecal assemblages disagrees with previous molecular evidence suggesting a feeding preference for Chaetocerotaceae diatoms [28]. This discrepancy may reflect differences in frustule robustness. Weakly silicified diatoms such as *Chaetoceros* may be more easily fragmented, making them difficult to identify microscopically, while their DNA remains detectable. These patterns are consistent with the idea that weakly silicified taxa may be more readily fragmented and/or dissolved during sinking through the water column [74], potentially contributing to their low detectability in feces. This theory is also supported by laboratory studies showing that juvenile growth is improved on diets containing weakly silicified diatoms (e.g., *Cylindrotheca fusiformis*) compared to microalgae with thicker cell walls [32]. Collectively, these findings suggest that juvenile *A. japonicus* can also utilize diatom cell contents via mechanical processing, while the mechanisms governing taxon-specific digestion and detectability remain to be clarified.

## 5. Conclusions

In this study, food availability and utilization of diatoms by juveniles of *A. japonicus* were investigated through field surveys and laboratory feeding experiments. The results of the field survey demonstrated that diatoms are one of the major food items for juvenile *A. japonicus* as well as adult individuals in a natural environment, although their relative importance as food varies seasonally with shifts in main food sources. Furthermore, this study suggests that diatom availability and digestibility for *A. japonicus* juveniles vary among species depending on ecological traits, and that *Tabularia* sp. was a particularly favorable food option due to its high availability and digestibility. These findings advance our understanding of optimal food sources for *A. japonicus* juveniles as well as feeding ecology in natural environments, thereby informing the selection of more suitable diets and potentially enhancing stock enhancement strategies.

## Figures and Tables

**Table 1:** (a) Ratio of diatom genera and (b) the proportions of non-viable cells in the feeding environments (water column, biofilm) and feces of *A. japonicus* juveniles from March to December 2019. Diatom taxa belonging to the same genus were grouped as “spp.” because individual morphotypes were not distinguished during the observations.

(a)	Taxa	Ratio (%)															
		Surface Water				Bottom Water				Biofilm				Feces			
		March	June	September	December	March	June	September	December	March	June	September	December	March	June	September	December
	Benthic																
	*Achnanthes* spp.											0.9		0.3		0.9	0.7
	*Amphiploura* spp.																0.3
	*Amphora* spp.	2.1			13.0		2.7			8.6	8.0	11.4	5.6	6.4	5.6	14.2	15.2
	*Biddulphia* spp.														0.5		
	*Cocconeis* spp.	4.1	4.3	2.1	21.7	4.1	2.7		18.2	14.3	11.5	17.5	4.0	7.7	9.3	6.2	15.8
	*Cylindrotheca* spp.	1.0				1.0											
	*Diploneis* spp.														0.3	2.7	0.7
	*Entomoneis* spp.				4.3				9.1					0.7	0.3	6.8	2.0
	*Grammatophora* spp.										1.1			0.3	0.3	0.9	1.0
	*Hantzschia* spp.														0.3		
	*Licmophora* spp.	1.0				2.0	2.7			1.0	1.1			2.0	5.1	1.8	2.4
	*Melosira* spp.	2.1												0.7	0.8	0.3	1.0
	*Navicula* spp.	5.2	1.4			1.0	5.5	5.6	36.4	13.3	14.9	11.4	8.9	21.5	15.7	18.1	23.9
	*Nitzschia* spp.	2.1	2.9	10.6	17.4	7.1	1.4	3.7	18.2	9.5	25.3	34.2	32.3	3.0	4.5	12.2	14.1
	*Odontella* spp.													0.3		1.2	4.4
	*Opephora* spp.	2.1	1.4		4.3					32.4	25.3	15.8	40.3	10.7	12.6	3.6	3.7
	*Plagiotropis* spp.														0.5		
	*Pleurosigma* spp.				4.3				9.1						0.3		
	*Rhoicosphenia* spp.											2.6	0.8		0.5	1.2	0.7
	*Stauroneis* spp.						1.4										
	*Tabularia* spp.	7.2	7.1		17.4	1.0			9.1	21.0	12.6	6.1	8.1	28.5	42.2	27.0	10.8
	Planktonic																
	*Chaetoceros* spp.			70.2		4.1	12.3	48.1								1.8	
	*Dactyliosolen* spp.	3.1	25.7	4.3		1.0	17.8										
	*Ditylum* spp.							3.7									
	*Guinardia* spp.					3.1										0.3	
	*Leptocylindrus* spp.		54.3		4.3		32.9	9.3									
	*Pseudo-nitzschia* spp.		2.9				12.3	16.7									
	*Skeletonema* spp.	7.2		12.8	13.0	7.1	8.2	9.3						1.0			
	*Thalassiosira* spp.	59.8				67.3		3.7						14.4			
	Unidentifiable																
	Centric diatoms													1.0	1.3	0.9	3.4
	Pennate diatoms	3.1				1.0								1.3	0.3		
**(b)**	**Taxa**	**Proportion of Non-Viable Cells (%)**															
		**Surface water**				**Bottom water**				**Biofilm**				**Feces**			
		**March**	**June**	**September**	**December**	**March**	**June**	**September**	**December**	**March**	**June**	**September**	**December**	**March**	**June**	**September**	**December**
	Benthic																
	*Achnanthes* spp.											0		0		100	100
	*Amphiploura* spp.																100
	*Amphora* spp.	100			100		50.0			33.3	14.3	69.2	0	68.4	100	85.4	93.3
	*Biddulphia* spp.														100		
	*Cocconeis* spp.	100	100	100	100	25.0	100		100	46.7	50.0	60.0	60.0	100	100	100	100
	*Cylindrotheca* spp.	0				0											
	*Diploneis* spp.														100	11.1	100
	*Entomoneis* spp.				0				0					50	100	34.8	33.3
	*Grammatophora* spp.										0			100	100	100	100
	*Hantzschia* spp.														100		
	*Licmophora* spp.	100				50.0	100			100	0			100	100	83.3	100
	*Melosira* spp.	0												0	0	0	0
	*Navicula* spp.	20.0	0			0	25.0	33.3	0	0	30.8	38.5	18.2	75	82.3	80.3	91.5
	*Nitzschia* spp.	50.0	50.0	0	25.0	57.1	100	0	0	20.0	45.5	17.9	10.0	66.7	61.1	65.9	81.0
	*Odontella* spp.													100		75.0	0
	*Opephora* spp.	50.0	0		100					14.7	59.1	27.8	8.0	100	100	100	100
	*Plagiotropis* spp.														100		
	*Pleurosigma* spp.				0				0						100		
	*Rhoicosphenia* spp.											0	0		100	100	100
	*Stauroneis* spp.						0										
	*Tabularia* spp.	28.6	40.0		75.0	0			0	36.4	63.6	14.3	10.0	95.3	96.4	93.4	100
	Planktonic																
	*Chaetoceros* spp.			0		0	0	0								100	
	*Dactyliosolen* spp.	0	0	0		0	0										
	*Ditylum* spp.							0									
	*Guinardia* spp.					0										100	
	*Leptocylindrus* spp.		0		0		0	0									
	*Pseudo-nitzschia* spp.		0				0	0									
	*Skeletonema* spp.	0		0	0	0	0	0						0			
	*Thalassiosira* spp.	0				0		0						34.9			
	Unidentifiable																
	Centric diatoms													66.7	40.0	66.7	70.0
	Pennate diatoms	0				0								100	100		

## Data Availability

The original contributions presented in this study are included in the article. Further inquiries can be directed to the corresponding authors.

## References

[1] Purcell S.W., Lovatelli A., González-Wangüemert M., Solís-Marín F.A., Samyn Y., Conand C. (2023). Commercially Important Sea Cucumbers of the World.

[2] Chen J., Chang Y., Brown N., Eddy S. (2015). Sea Cucumber Aquaculture in China. Echinoderm Aquaculture.

[3] Oh G., Ko S., Lee D.H., Heo S., Jung W. (2017). Biological activities and biomedical potential of sea cucumber (*Stichopus japonicus*): A review. Fish. Aquat. Sci..

[4] Purcell S.W., Williamson D.H., Ngaluafe P. (2018). Chinese market prices of bêche-de-mer: Implications for fisheries and aquaculture. Mar. Policy.

[5] Purcell S.W., Shea S.K.H., Gray B.C.T. (2025). Decadal changes in value of dried sea cucumber (bêche-de-mer) in Hong Kong markets. Mar. Policy.

[6] Toral-Granda V., Lovatelli A., Vasconcellos M. (2008). Sea Cucumbers: A Global Review of Fisheries and Trade.

[7] Hamel J.-F., Mercier A. (2013). *Apostichopus japonicus*. The IUCN Red List of Threatened Species 2013, 2013, e.T180424A1629389. IUCN.

[8] Uthicke S., Welch D., Benzie J.A.H. (2004). Slow growth and lack of recovery in overfished holothurians on the Great Barrier Reef: Evidence from DNA fingerprints and repeated large-scale surveys. Conserv. Biol..

[9] Purcell S.W., Lovatelli A., Conand C., Purcell S., Uthicke S., Hamel J.F., Mercier A. (2004). Criteria for release strategies and evaluating the restocking of sea cucumbers. Advances in Sea Cucumber Aquaculture and Management.

[10] Bruckner A.W., Johnson K.A., Field J.D. (2003). Conservation strategies for sea cucumbers: Can a CITES Appendix II listing promote sustainable international trade?. SPC Bêche-De Mer Inf. Bull..

[11] Sakai Y. (2015). Mass production of artificial seed for the Japanese common sea cucumber (*Apostichopus japonicus*) in Hokkaido, Japan. Bull. Fish. Res. Agen..

[12] Ministry of Agriculture, Forestry and Fisheries, Japan. https://www.maff.go.jp.

[13] Hokkaido Prefectural Government. https://www.pref.hokkaido.lg.jp.

[14] Furukawa N., Furukawa Y., Yamana Y., Kashio S., Uekusa R., Goshima S. (2016). Growth and survival of released juveniles of the Japanese sea cucumber *Apostichopus japonicus* into artificial reef. Bull. Fish. Sci. Hokkaido Univ..

[15] Tanaka M. (2000). Diminution of sea cucumber *Stichopus japonicus* juveniles released on artificial reefs. Ishikawa Prefect. Fish. Res. Center.

[16] Kusaka K., Izumikawa K., Ikeda Z. (1995). Technical developments in the release of artificial seedlings of sea cucumber *Stichopus japonicus*. Bull. Fish. Exp. Stn. Okayama Prefect..

[17] Hatanaka H., Uwaoku H., Yasuda T. (1994). Experimental studies on the predation of juvenile sea cucumber, *Stichopus japonicus* by sea star, *Asterina peclinifera*. Suisan Zoshoku.

[18] Inaba N., Matsumoto T., Kawai H., Anaguchi Y., Matsuno K. (2021). Predation of juvenile sea cucumber *Apostichopus japonicus* by kelp crab *Pugettia ferox*. Front. Mar. Sci..

[19] Inaba N., Matsumoto T., Anaguchi Y., Matsuno K. (2025). Mortality risk of juvenile sea cucumber *Apostichopus japonicus* by the sympatrically occurring hermit crab *Paguristes orttmani*. Aquac. Res..

[20] Xu Q., Hamel J.-F., Mercier A., Yang H., Hamel J.-F., Mercier A. (2015). Feeding, digestion, nutritional physiology, and bioenergetics. The Sea Cucumber Apostichopus japonicus: History, Biology and Aquaculture; Developments in Aquaculture and Fisheries Science.

[21] Cheo S. (1963). Study of Sea Cucumber: Morphology, Ecology and Propagation of Sea Cucumber.

[22] Gao F. (2008). Seasonal Variations of Nutritional Composition, Food Resources, and Digestive Physiology in Sea Cucumber *Apostichopus japonicus*. Doctor Thesis.

[23] Gao F., Xu Q., Yang H. (2010). Seasonal variations of food sources in *Apostichopus japonicus* indicated by fatty acid biomarkers analysis. J. Fish. China.

[24] Sun Z., Gao Q., Dong S., Paul K.S.S., Wang F. (2013). Seasonal changes in food uptake by the sea cucumber *Apostichopus japonicus* in a farm pond: Evidence from C and N stable isotopes. J. Ocean Univ. China.

[25] Wen B., Gao Q.-F., Dong S.-L., Hou Y.-R., Yu H.-B., Li W.-D. (2016). Uptake of benthic matter by sea cucumber *Apostichopus japonicus* (Selenka): Insights from carbon stable isotopes and fatty acid profiles. J. Exp. Mar. Biol. Ecol..

[26] Gao F., Li F.H., Tan J., Yan J.P., Sun H.L. (2014). Bacterial community composition in the gut content and ambient sediment of sea cucumber *Apostichopus japonicus* revealed by 16s rRNA gene pyrosequencing. PLoS ONE.

[27] Zhang H., Xu Q., Zhao Y., Yang H. (2016). Sea cucumber (*Apostichopus japonicus*) eukaryotic food source composition determined by 18s rDNAbarcoding. Mar. Biol..

[28] Yamazaki Y., Sakai Y., Mino S., Sawabe T. (2020). An annual faecal 16s amplicon sequencing of individual sea cucumber (*Apostichopus japonicus*) demonstrates the feeding behaviours against eukaryotes in natural environments. Aquac. Res..

[29] Shiell G. (2004). Field observations of juvenile sea cucumbers. SPC Bêche-De Mer Inf. Bull..

[30] Yamana Y., Furukawa Y., Kashio S., Goshima S. (2014). Environmental characteristics of the habitats of juvenile sea cucumber *Apostichopus japonicus* around Hokkaido—Several inferences from southern Hokkaido. Aquac. Sci..

[31] Han Q., Keesing J.K., Liu D. (2016). A review of sea cucumber aquaculture, ranching, and stock enhancement in China. Rev. Fish. Sci. Aquac..

[32] Shi C., Dong S., Wang F., Gao Q., Tian X. (2013). Effects of four fresh microalgae in diet on growth and energy budget of juvenile sea cucumber *Apostichopus japonicus* (Selenka). Aquaculture.

[33] Shi C., Dong S., Wang F., Gao Q., Tian X. (2015). Effects of the sizes of mud or sand particles in feed on growth and energy budgets of young sea cucumber (*Apostichopus japonicus*). Aquaculture.

[34] Xie X., Zhao W., Yang M., Zhao S., Wei J. (2017). Beneficial effects of benthic diatoms on growth and physiological performance in juvenile sea cucumber *Apostichopus japonicus* (Selenka). Aquac. Int..

[35] Hamm C.E., Merkel R., Springer O., Jurkojc P., Maler C., Prechtel K., Smetacek V. (2003). Architecture and material properties of diatom shells provide effective mechanical protection. Nature.

[36] Pančić M., Torres R.R., Almeda R., Kiørboe T. (2019). Silicified cell walls as a defensive trait in diatoms. Proc. R. Soc. B.

[37] Massin C., Jangoux M., Lawrence J.M. (1982). Food and feeding mechanisms: Holothuridea. Echinoderm Nutrition.

[38] Ezaki Y. (2001). Suitable rearing conditions for stable production of young Japanese sea-cucumber. Bull. Fukuoka Fisheries Mar. Technol. Res. Cent..

[39] Holm-Hansen O., Lorenzen C.J., Holmes R.W., Strickland J.D.H. (1965). Fluorometric determination of chlorophyll. ICES J. Mar. Sci..

[40] Jin D., Cheng Z., Lin J., Liu S. (1985). The Marine Benthic Diatoms in China Volume I.

[41] Hasle G.R., Syvertsen E.E., Tomas C.R. (1997). Marine Diatoms. Identifying Marine Phytoplankton.

[42] Williams D.M., Round F.E. (1986). Revision of the genus *Synedra* Ehrenb. Diatom Res..

[43] Kuriyama K., Suzuki H., Nagumo T., Tanaka J. (2010). Morphology and taxonomy of marine benthic diatom (1), *Tabularia* (*Fragilariaceae*, *Fragilariales*). J. Jpn. Bot..

[44] Nishida C., Kijima K., Sakai M., Kawakami M., Amada K. (2016). Morphology of *Tabularia tabulata* from the Hakata River, Fukuoka Prefecture, Western Japan and identification key to *Tabularia* species (*Fragilariaceae*). J. Jpn. Bot..

[45] Tokushima H., Inoue-Kashino N., Nakazato Y., Masuda A., Ifuku K., Kashino Y. (2016). Advantageous characteristics of the diatom *Chaetoceros gracilis* as a sustainable biofuel producer. Biotechnol. Biofuels.

[46] Khaw Y.S., Yusoff F.M., Tan H.T., Noor Mazli N.A.I., Nazarudin M.F., Shaharuddin N.A., Omar A.R., Takahashi K. (2022). Fucoxanthin Production of Microalgae under Different Culture Factors: A Systematic Review. Mar. Drugs.

[47] Liu L., Du R., Zhang X., Dong S., Sun S. (2017). Succession and seasonal variation in epilithic biofilms on artificial reefs in culture waters of the sea cucumber *Apostichopus japonicus*. J. Oceanol. Limnol..

[48] Fukui D., Kitatsuji S., Ikeda T., Shiga N., Yamaguchi A. (2010). Long-term changes in the abundance and community structure of net-phytoplankton in Oshoro Bay, Hokkaido. Bull. Plankton Soc. Japan.

[49] Matsumoto T., Matsuno K., Katakura S., Kasai H., Yamaguchi A. (2021). Seasonal variability of the protist community and production in the southern Okhotsk Sea revealed by weekly monitoring. Reg. Stud. Mar. Sci..

[50] Hamao Y., Morimoto K., Tatamisashi S., Wakita M., Kasai A., Matsuno K. (2024). Seasonal changes in the protist communities of Hakodate Bay, southern Hokkaido, from 2020 to 2022. Reg. Stud. Mar. Sci..

[51] Lari E., Steinkey D., Morandi G., Rasmussen J.B., Giesy J.P., Pyle G.G. (2017). Oil sands process-affected water impairs feeding by *Daphnia magna*. Chemosphere.

[52] Sathish K., Patil J.S., Anil A.C. (2020). Phytoplankton chlorophyll-breakdown pathway: Implication in ecosystem assessment. J. Environ. Manag..

[53] David F., Raymond G., Grys J., Ameziane N., Sadoul B. (2024). Survival, growth, and food resources of juvenile sea cucumbers *Holothuria forskali* (Echinodermata, Holothuroidea) in co-culture with sAuahellfish in Brittany (France). Aquac. Nutr..

[54] Kurogi M., Akiyama K. (1957). Studies on ecology and culture of *Undaria pinnatifida* (Sur.) Hariot. Bull. Tohoku Reg. Fish. Res. Lab..

[55] Funano T. (1983). The ecology of *Laminaria religiosa* MIYABE I. The life history and the alternation of nuclear phases of *Laminaria religiosa*, and the physiological ecology of the gametophytes and the embryonal sporophytes. Sci. Rep. Hokkaido Fish. Exp. Stn..

[56] Gao H., Endo H., Agatsuma Y. (2015). Effect of increased seawater temperature on biomass, growth, and maturation of *Saccharina japonica* near its southern limit in northern Japan. J. Appl. Phycol..

[57] Japan Meteorological Agency. https://www.jma.go.jp/jma/index.html.

[58] Michio K., Kengo K., Yasunori K., Hitoshi M., Takayuki Y., Hideaki Y., Hiroshi S. (2003). Effects of deposit feeder *Stichopus japonicus* on algal bloom and organic matter contents of bottom sediments of the enclosed sea. Mar. Pollut. Bull..

[59] Pierrat J., Bédier A., Eeckhaut I., Magalon H., Frouin P. (2022). Sophistication in a seemingly simple creature: A review of wild holothurian nutrition in marine ecosystems. Biol. Rev..

[60] Slater M.J., Jeffs A.G., Sewell M.A. (2011). Organically selective movement and deposit-feeding in juvenile sea cucumber, *Australostichopus mollis* determined in situ and in the laboratory. J. Exp. Mar. Biol. Ecol..

[61] Passy S.I. (2007). Diatom ecological guilds display distinct and predictable behavior along nutrient and disturbance gradients in running waters. Aquat. Bot..

[62] Beldowska M., Zgrundo A., Kobos J. (2018). Mercury in the diatoms of various ecological formations. Water Air Soil Pollut..

[63] Rimet F., Bouchez A. (2012). Live-forms, cell-sizes and ecological guilds of diatoms in European rivers. Knowl. Manag. Aquat. Ecosyst..

[64] Uthicke S. (1999). Sediment bioturbation and impact of feeding activity of *Holothuria* (*Halodeima*) *atras* and *Stichopus chloronotus*, two sediment feeding holothurians, at Lizard Island, Great Barrier Reef. Bull. Mar. Sci..

[65] Sonnenholzner J. (2003). Seasonal variation in the food composition of *Holothuria theeli* (Holothroidea: Aspidochirotida) with observations on density and distribution patterns at the central coast of Ecuador. Bull. Mar. Sci..

[66] Sullivan B.K., Miller C.B., Peterson W.T., Soeldner A.H. (1975). A scanning electron microscope study of the mandibular morphology of boreal copepods. Mar. Biol..

[67] Suh H. (1996). The gastric mill of euphausiid crustaceans: A comparison of eleven species. Hydrobiologia.

[68] Huang X., Xie R., Zou A., Zhang S., Xu X., Sun G., Yang J. (2024). Effects of polypeptidin feeding on growth and intestinal flora of *Apostichopus japonicus*. Aquac. Rep..

[69] Li W., Xiao H., Li S., Zhang J., Deng Y., Liu Z., Han L., Sun J., Chang Y., Ding J. (2025). Effects of dietary distiller’s grains on growth and gut microbiota of the sea cucumber *Apostichopus japonicus*. Aquac. Rep..

[70] Pan W., Wang X., Ren C., Jiang X., Gong S., Xie J., Wong N.K., Li X., Huang J., Fan D. (2024). Sea cucumbers and their symbiotic microbiome have evolved to feed on seabed sediments. Nat. Commun..

[71] Zhou Y., Zhang J., Wang L., Xu H., Lin Z., Liu Y., Hao Z., Ding J., Chang Y. (2022). Characterization of the bacterial community in the ecosystem of sea cucumber (*Apostichopus japonicus*) culture ponds: Correlation and specificity in multiple media. Water.

[72] Zhang J., Zhou Y., Wang L., Liu Y., Lin Z., Hao Z., Ding J., Chang Y. (2022). Asymmetry evaluation of sea cucumber (*Apostichopus japonicus*) gut and its surrounding environment in the bacterial community. Symmetry.

[73] Houki S., Kawamura T., Ogawa N., Watanabe Y. (2018). Efficient crushing of hard benthic diatoms in the gut of the Manila clam *Ruditapes philippinarum*-Experimental and observational evidence. J. Exp. Mar. Biol. Ecol..

[74] Pandey M., Biswas H., Schmidt S. (2025). Distribution of organic matter and diatom frustules (diversity, flux) along the western Indian continental shelf related to contrasting physicochemical settings. Mar. Environ. Res..

